# Associations between APOE genotype and cerebral small-vessel disease: a longitudinal study

**DOI:** 10.18632/oncotarget.17724

**Published:** 2017-05-09

**Authors:** Xiao Luo, Yerfan Jiaerken, Xinfeng Yu, Peiyu Huang, Tiantian Qiu, Yunlu Jia, Kaicheng Li, Xiaojun Xu, Zhujing Shen, Xiaojun Guan, Jiong Zhou, Minming Zhang, for The Alzheimer’s Disease Neuroimaging Initiative (ADNI)

**Affiliations:** ^1^ Department of Radiology, The Second Affiliated Affiliated Hospital of Zhejiang University School of Medicine, Hangzhou, China; ^2^ Department of Neurology, The Second Affiliated Affiliated Hospital of Zhejiang University School of Medicine, Hangzhou, China; ^3^ Department of Surgical Oncology, Sir Run Run Shaw Hospital, College of Medicine, Zhejiang University, Hangzhou, China

**Keywords:** apolipoprotein E (APOE), cerebral small-vascular disease (CSVD), white matter hyperintensities (WMH), dilated perivascular space (dPVS), cognition

## Abstract

**Objective:**

It remains unclear if and how the interactions between APOE genotypes and cerebral small-vessel diseases (CSVD) lead to cognitive decline in the long term. Based on ADNI cohort, this longitudinal study aimed to clarify the potential relationship among APOE genotype, CSVD and cognition by integrating multi-level data.

**Method:**

There were 135 healthy elderly (including ε2, ε4 allele carriers and ε3 homozygotes) who had completed two years’ follow-up. MRI markers of CSVD, including white matter hyperintensities (WMH), dilated perivascular space (dPVS), microbleeds and lacune, were assessed. Besides, neuropathological factors including Alzheimer's disease-related pathology measured by CSF and PiB-PET were assessed. Repeated measurements ANOVAs were performed to test impact of different APOE genotypes on CSVD.

**Results:**

We found that APOE ε4 carriers had significantly more frontal WMH burden and basal ganglia dPVS at baseline and faster progression of frontal WMH burden during follow-up. Furthermore, our results showed that APOE ε4 carriers had significantly decreased Aβ_1-42_ level, and its level was negatively related with baseline and progressive total WMH burden. Then, general linear modals indicated interaction between basal frontal WMH burden and ε4 allele was related with declining trend of cognition.

**Conclusion:**

Our findings suggested APOE ε4 allele was associated with increased Aβ deposition, which may lead to the formation and progression of WMH, especially in frontal lobe. Besides, interaction between the increased frontal WMH burden and ε4 allele can exert long-term detrimental effects on individual's trajectory of cognition.

## INTRODUCTION

Cerebral small-vascular diseases (CSVD) are common accompaniments of aging and they could be assessed non-invasively via conventional MRI. According to the latest neuroimaging standards, CSVD include white matter hyperintensities (WMH), dilated perivascular space (dPVS), microbleeds (MBs) and lacune [1, 2]. There are evidences that they often co-occur and presence of them can increase the future risk of cognitive decline and dementia [3, 4]. Although mechanisms underlying association between CSVD and cognition are unclear, previous studies hypothesized that cerebral small-vessel burden may expedite progression of cognitive-related diseases through promoting accumulations of amyloid depositions or increasing vulnerability to these pathology [5–7]. Another known risk factor for age-related cognitive decline is in the APOE gene loci. It is known that APOE is involved in modulating the metabolism and accumulation of amyloid depositions [8, 9]. However, the precise mechanisms underlying its relationship with the risk of cognitive deficits are also not fully understood.

Clarifying the relationship between APOE genotype and CSVD could provide important clues to the mechanisms underlying the cognitive impairment. Notably, two high-quality meta-analyses reported inconsistent results regarding the association between APOE ε2/ε3/ε4 genotypes and CSVD in older adults. The work of Paternoster et al. (24 studies, n=8546) suggested there was no evidence of association between APOE ε4 allele and WMH [10]. Whereas meta-analysis of Schilling et al. (11 studies, limited to use continuous WMH burden, n=8917) displayed that APOE e4 carrying was significantly associated with increased WMH and presence of MBs, and APOE ε2 allele was significantly related with increasing WMH load [11]. Notably, most studies selected in these meta-analyses are cross-sectional and performed in patients with dementia, depression or other diseases, these limitations may contribute to the discrepancy. Thus, longitudinal studies are needed to determine whether different APOE genotypes are associated with CSVD in the general population. Moreover, studies examining neuropathologic correlates of CSVD and their association with APOE genotypes could also help in understanding the pathophysiologic link between APOE and CSVD.

The current longitudinal study is aim to dissect the relationship between APOE genotype and CSVD in a two-years cohort of healthy elderly from ADNI database, and to explore whether there are interactions between APOE and CSVD lead to cognitive decline. Specifically, in three time points, lobar distribution of WMH volume was quantified, dPVS were observed in the basal ganglia (BG) and white matter (WM) region, and number of MBs and lacune was calculated. Besides, dementia-related neuropathologic biomarkers measured by CSF or neuroimaging were also taken into consideration in this study. First, we predicted that e4 allele was associated with formation and progression of WMH, especially in brain regions with perilous blood supply. Second, we hypothesized that the synergy between ε4 allele and CSVD can exert long term detrimental effects on cognition.

## RESULTS

### Demographics, behavioral and CSF data

An overview of demographic variables, behavioral performance and CSF data is presented in Table 1. There were no significant differences in age, gender, or education among three groups (P>0.05). Additionally, there was no significant difference in cardiovascular risk profile among groups as determined by HIS, hypertension, smoking history and diabetes. In current study, the ε2, ε3, and ε4 allele have approximate frequencies of 14.8%, 54.8% and 30.4%. Notably, the percentage of ε3 homozygotes was far below the figure in a healthy Caucasian population (about 80% [19]). This inconsistence may attribute to subjects who carrying ε4 allele may more likely to receive follow-up examination.

**Table 1:** Comparison among three subgroups of demographic, behavior and CSF data

	ε2 carriers	ε4 carriers	Controls	F/χ^2^	P
Number	20	41	74		
Demographic characteristics					
Age, y, mean (SD)	73.57±5.21	73.33±7.11	74.26±6.12	0.31	0.73
Female, n(%)	10 (50.0%)	20 (48.8%)	34 (45.9%)	0.15	0.93
Education, y, mean (SD)	16.85±2.50	16.20±2.40	16.80±2.41	0.92	0.40
Vascular risk factors, n(%)					
Hypertension	11 (55.0%)	19 (46.3%)	36 (48.6%)	0.41	0.82
Smoking history	14 (70.0%)	30 (73.2%)	59 (79.7%)	1.14	0.57
Diabetes	19 (95.0%)	39 (95.1%)	67 (90.5%)	1.01	0.61
Hachinski Ischemic Score	0.75±0.91	0.46±0.50	0.55±0.64	1.30	0.28
CSF biomarker					
Amyloid_1–42_ (ng/L)	223.57±50.34	171.33±55.50	204.68±47.47	7.08	<0.001
T-tau (ng/L)	55.64±22.13	71.25±31.16	71.25±40.16	1.56	0.22
P-tau_181_ (ng/L)	34.16±16.05	36.71±15.93	36.22±26.10	0.07	0.93
Cognitive scores					
MMSE_baseline	28.70±1.38	28.90±1.28	29.31±1.02	3.00	0.05
MMSE_1 year follow-up	29.20±1.01	28.73±1.55	28.80±1.25	0.92	0.40
MMSE_2 year follow-up	29.05±1.05	28.63±1.85	28.95±1.32	0.77	0.47

MMSE: Mini-Mental State Examination; *represent P < 0.05

It should be noted that mean level of Aβ_1-42_, t-tau and p-tau_181_ levels in Table 1 only represent the subjects who had CSF sample.

As for CSF data, we observed APOE ε4 carriers had significantly decreased concentration of Aβ_1-42_ compared with both APOE ε2 carriers and controls (p<0.01). The difference was still significant after adjusting for age and sex factors (p<0.01). However, no significant differences of level of t-tau and p-tau_181_ were found.

### Group differences of CSVD at baseline and during follow-up

At baseline, there were significant differences in total WMH (p<0.05) and frontal WMH volume (p<0.05) among 3 groups, the differences were still significant after adjusting for age and sex (total WMH volume p=0.22, frontal WMH volume p=0.15); further post-hoc t-tests revealed that there were significantly increased total WMH and frontal WMH volume in APOE ε4 carriers when compared to controls (p<0.05, corrected by Bonferroni); in contrast, no differences of WMH volume between APOE ε2 carriers and any other group were found. Furthermore, after adding Aβ_1-42_ concentration as co-variants, the group differences of WMH cease to exist, while Aβ_1-42_ concentration becomes the most predominant factor influencing WMH volume (P<0.05)

The non-parametric K-W test of severity of dPVS revealed that there are significant differences in dPVS in BG (p<0.01) among three groups, and post-hoc analyses showed APOE ε4 carriers had more severity of dPVS in BG than controls (p<0.01), but there were no significant differences between APOE ε2 carriers and any other groups. When controlling for total WMH volume as covariance, it should be noted that difference of BG dPVS between APOE ε4 carriers and controls was no longer significant. As for dPVS in WM region, there was no significant difference. With regard to MBs and lacune, no significant differences were found among three groups at baseline.

**Figure 1:**
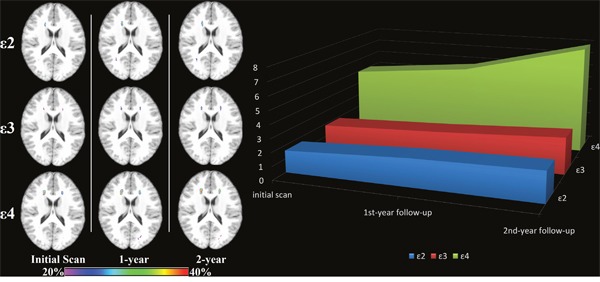
Progression of WMH volume during 2 years follow-up Only the progression of frontal WMH volume and progression of total WMH volume had significant between-groups differences (p<0.05, Unit: ml).

ANOVA of repeated measures data results revealed that WMH volume increased during 2 year follow-up in all lobe but only in frontal lobe the AOPE group differences had significant impact on the progression of WMH volume (p<0.05). The difference was significant after adjusting for age and sex (p=0.22). Tests also revealed that lacune, MB and dPVS progressed during the 2-year follow-up but their progression had no significant group difference neither (Figure 1).

### Correlation between CSF data and CSVD

**Figure 2:**
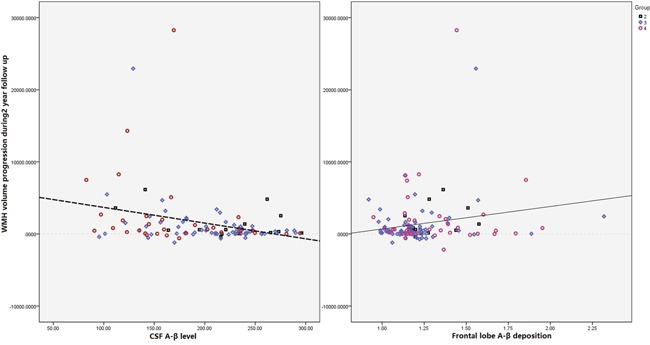
Scatter plot diagram of correlation between progression of total WMH (unit: ml) and neuropathological data (measured by CSF and PiB PET) Our results showed that both Aβ in CSF (R=-0.0224, P=0.011) and PiB-PET assessed Aβ deposition in frontal lobe (R=-0.185, P=0.032) were significantly correlated with progression of WMH volume.

Correlation tests showed that concentration of Aβ_1-42_ is correlation with WMH volume (r=-0.254, P<0.01), progression of WMH volume (r=-0.295, P<0.005) and dPVS in white matter area (r=-0.257, P<0.005, Figure 2). But there were no significant correlation of dPVS in basal ganglia, lacune and MB with concentration of Aβ_1-42_.

### Correlation between cognition and CSVD

Across all study subjects, baseline MMSE ranking was not correlated with any CSVD index, and progression of MMSE was correlated with progression of WMH in frontal lobe (r= -0.198, p<0.05). In subgroup level, neither baseline MMSE nor progression of MMSE was correlated with any CSVD index in ε2 carriers and controls. However, we found that progression of MMSE was significantly related with baseline frontal lobe WMH volume (r= -0.316, p<0.05) and progression of frontal lobe WMH volume (r=-0.47, p<0.00) in APOE ε4 carriers.

Also, progression of MMSE is correlated with baseline dPVS severity in BG (r=-0.43, p<0.01), but no longer significant after controlling for total WMH volume. With regard to MBs and lacune, no such significant correlations were found.

Finally, variables entered into the GLM models are: baseline frontal lobe WMH volume, lacune, dPVS in basal ganglia and centrum ovale, E2 and E4 gene carrying status, HBP score, age, smoking history, diagnosed diabetes mellitus, and all the 2-way interaction between gene status frontal lobe WMH volume, lacune and dPVS. GLM indicated the independent factors impacting MMSE ranking were the interaction between APOE ε4 allele and frontal WMH volume (Type III sum of square=9.011, p=0.05, Table 2) and the interaction between APOE ε2 allele and number of lacunar (Type III sum of square=9.135, p=0.049, Table 2).

**Table 2:** Tests of between-subjects effects dependent variable: MMSE score progression

Source	Type III sum of squares	Significant
Age	6.37	0.099
Sex	0.017	0.932
APOE ε2 gene carrying	0.162	0.791
APOE ε4 gene carrying	0.539	0.63
Frontal lobe WMH volume	0.085	0.848
Number of lacunes	0.56	0.623
dPVS grade in centrum ovale	1.901	0.366
dPVS in basal ganglia	0.406	0.676
HBP score	0.089	0.844
APOE ε2 × dPVS in basal ganglia	0.271	0.732
APOE ε4 × dPVS in basal ganglia	0.011	0.945
APOE ε2 × dPVS in centrum ovale	1.186	0.475
APOE ε4 × dPVS in centrum ovale	0.002	0.98
APOE ε2 × Frontal lobe WMH volume	0.605	0.609
APOE ε4 × Frontal lobe WMH volume	9.011	0.050*
APOE ε2 × lacune	9.135	0.049*
APOE ε4 × lacune	0.203	0.844

P<0.05 was considered statistical significant, the factors influencing MMSE progression.

## DISCUSSION

In present longitudinal study, we mainly observed that 1) APOE ε4 carriers had significantly decreased concentration of Aβ_1-42_ and increased frontal PiB uptake value relative to ε2 carriers and controls; 2) APOE ε4 allele was associated with increasing frontal WMH volume and severity of BG PVR at baseline; moreover, significant progression of frontal WMH volume in APOE ε4 carriers relative to ε2 carriers and controls; 3), in ε4 carriers, both the baseline frontal WMH volume and progression of frontal WMH volume was related with cognitive decline.

### Relationship between APOE and WMH

At baseline, we observed that APOE ε4 carriers had significantly more frontal WMH volume. Moreover, our results showed APOE ε4 carriers had decreased CSF Aβ_1-42_ level and increased PET-PiB SUV in frontal lobe relative to controls. On the one hand, physiological interpretations of our observations may be that the vulnerability of frontal WM to ischemic condition [20, 21]. We noted that most frontal WMH is distributed around the anterior periventricular horn. Studies have found a watershed area around the periventricular region where blood supply is perilous. To be specific, anastomoses in this area are scarce and arterioles are tortuous, thus making the WM in anterior periventricular horn extra vulnerable to hypoperfusion and small vessel disease [22]. On the other hand, APOE ε4 allele is associated with aggregation of Aβ protein and decreased clearance of Aβ deposition relative to ε3 or ε2 allele [23, 24]. According to latest studies, possible mechanisms include ε4 allele is associated with sleep disorders [25], further, lead to impaired glymphatic clearance and suppressed clearance of soluble amyloid deposits [26–29]. As a result, relatively increased Aβ deposition in ε4 carriers may damage arterial wall and reduce vessel lumen, leading to chronic hypoperfusion [30]. Taken together, we speculated that perilous blood supply in frontal lobe and accumulation of mis-folded protein aggregates jointly contribute to formation of WMH.

Longitudinally, we found progression of frontal WMH volume in APOE ε4 group was also significantly faster than the two other groups. These results suggested APOE ε4 allele has long-term detrimental effects on formation of WMH, especially in frontal lobe, even in non-demented individuals. We assumed significantly increased frontal WMH burden in ε4 carriers at baseline may explain these results. On its own, WMH burden could reflect worse cerebral vascular condition, as studies has pointed out that WMH is correlated with reduced cerebral perfusion both inside WMH area and areas of normal appearing WM (i.e., penumbra) [31]. In these normal appearing WM areas surrounding WMH, many DTI studies have reported subtle damage already exists [32, 33]. These penumbra regions with tortuous blood vessel and subtle microstructural damage can easily develop into future WMH areas. Meanwhile, studies of hemodynamics further reported that the size of ischemic penumbra was larger than the one assessed by microstructural modalities, suggesting WMH is progressively spreading to normal appearing WM, and putting more WM tracts at risk [34]. Moreover, our results showed that decreased Aβ_1-42_ level and increased PET-PiB SUV of frontal lobe at baseline were also significantly related with progression of frontal WMH volume. These may suggest Aβ deposition of subjects have a long-term effect on the formation of WMH.

Consistent with our hypothesis, results in this part suggested carrying ε4 allele was associated with WMH in non-demented subjects. We speculated that a combination of perilous blood supply in anterior periventricular horn and aggravated chronic hypoperfusion (result from increased Aβ deposition in APOE ε4 carriers) caused increased frontal WMH at baseline. Subsequently, these frontal WMHwould continuously expand owe to deteriorated cerebral vascular condition.

Correlation analyses showed that in ε4 carriers, baseline and progression of frontal WMH volume was related with declining trend of cognition in ε4 carriers. Furthermore, GLM indicated factors impacting MMSE ranking were the interaction between APOE ε4 allele and frontal WMH volume, independent to vascular risk factors (hypertension, diabetes mellitus and smoking history) and other CSVD (Table 2). Previous studies have found the correlation of reduced WM integrity in frontal lobe with cognitive decline [35, 36]. In particular, previous anatomical studies suggested the fronto-subcortical circuit play a critical part in the cognitive process such as planning and execution of self-generated novel action [37, 38]. Thus, we assumed that the significantly increased frontal WMH volume in non-demented ε4 carriers, which partially reflected the disruption of fronto-subcortical WM circuit, caused the damage to general cognition. These results were in line with our hypothesis that e4 allele can activate WMH to exert long-term detrimental effects on subjects’ cognition.

### Relationship between APOE and dPVS

In this section, our results showed that dPVS in BG area was increased in APOE ε4 carriers relative to controls, but there were no differences in WM dPVS or progression of dPVS. Previous studies have found that WM-dPVS is more closely related to Aβ accumulation and CAA pathology [39], while BG-dPVS preferentially associated with hypertension [40]. However in our study, there were no significance increases in WM-dPVS in APOE ε4 group where we presumed the Aβ accumulation should be more prominent. According to the evidence that dPVS is closely related to the severity of WMH [41], we thus assumed that increased BG-dPVS in ε4 carriers may mainly result from increased WMH burden in ε4 carriers. To validate this assumption, correlation analyses were performed. Results displayed that not only dPVS was correlated with WMH volume, but also the differences of BG-dPVS among groups were no longer significant after controlling for total WMH volume. Overall, our results may also suggest in non-demented elderly, dPVS is not directly involved with Aβ accumulation; therefore, future studies controlling WMH are required to determine the pathology associations for regional dPVS. Besides, longitudinal result in our analysis showed that there were no significant difference between progression of dPVS across groups. It suggested in non-demented elderly, the differences of APOE genotype only have limited impact on the development of dPVS.

Correlation analyses suggested baseline BG-dPVS was related with progression of general cognition; however, when total WMH volume was introduced into GLM, this association is no longer statistically significant. General enlargement of dPVS is associated with other morphological features of CSVD such as WMH or lacune [42, 43]. The association between WMH and cognitive decline has been extensively studied and WM tract damage has been considered an important pathological change in cognitive decline. In contrast, few studies focused on the pathological correlation between dPVS and cognitive decline. In general population, our results were in perfect accordance with one work, which documenting correlation between BG-dPVS and cognition is no longer significant after controlling for WMH volume [44]. Thus, we assumed that the correlation between dPVS and cognitive decline may be dependent on WM damage. However, it is worth noticing that in both our study and the work of Yicheng et al., the assessment of dPVS was based on a 4-grade semi-quantitative visual scales. Compared to the volumetric assessment of WMH, such grading system may be less sensitive. The correlation between dPVS and cognition are require further studied, but our results suggested WMH volume is a better radiological marker to reflect CSVD.

### Relationship between APOE and MBs/Lacune

There was no difference of number of MBs/Lacune among three groups at baseline or during two-year follow-up; moreover, there was still no association between MBs/lacune and cognition changes. In previous studies, the association between APOE ε2 allele and MBs has been reported. Some reasons may account for this discrepancy. On the one hand, the prevalence of MB and lacune in our population are 20.9% and 31.9% respectively, which is similar to the previous reported prevalence rate in general population [45]; however, those studies documenting associations between ε2 allele and MBs were mostly performed in mild cognitive impairment, dementia or stroke patients, who generally have higher prevalence of MBs and lacune. Therefore, relatively low prevalence rate of MBs and lacune in non-demented population may reduce the chance of finding a significant association. On the other hand, limitation in assessment methods in current analyses, such as calculating number of MBs based on T2*-weighted images, may also impact the outcome.

Conclusively, the results of our population-based cohort study emphasized that APOE ε4 allele was related with relative increased Aβ deposition and had a long-term detrimental effect on CSVD, especially in frontal WMH. Further, there is interaction between APOE ε4 allele and frontal WMH, which may exert long-term detrimental effects on non-demented individuals’ trajectory of cognitive change.

## MATERIALS AND METHODS

### Alzheimer's Disease Neuroimaging Initiative

Data set used in this study was obtained from the Alzheimer's disease Neuroimaging Initiative (ADNI) database (adni.loni.usc.edu). The ADNI was launched in 2003 by the National Institute on Aging (NIA), the National Institute of Biomedical Imaging and Bioengineering (NIBIB), the Food and Drug Administration (FDA), private pharmaceutical companies and non-profit organizations, as a $60 million, 5-year public-private partnership. The primary goal of ADNI has been to test whether serial magnetic resonance imaging (MRI), positron emission tomography (PET), other biological markers, and clinical and neuropsychological assessment can be combined to measure the progression of mild cognitive impairment (MCI) and early Alzheimer's disease (AD). Determination of sensitive and specific markers of very early AD progression is intended to aid researchers and clinicians in developing new treatments and monitor their effectiveness, as well as lessen the time and cost of clinical trials.

### Study participants

This study was approved by the Institutional Review Boards of all of the participating institutions, and informed written consent was obtained from all participants at each site. Imaging data and neuropsychological assessment data for each subject was gathered at 3 time points (baseline, 1 year and 2 years follow-up). Individuals who carrying at least one APOE ε4 allele (genotype ε4/ε4 or ε4/ε3) were classified as APOE ε4 carriers, who carrying at least one ε2 allele (genotype ε2/ε2 or ε2/ε3) were classified as APOE genotype ε2 carriers. Individuals with APOE genotype ε3/ε3 were classified as the controls in current study. Given the possible confounding effects of APOE-related effect, individuals with the APOE genotype ε2/ε4 were exclude [9]. Using the ADNI GO and ADNI 2databases, 147 cognitively intact healthy participants were identified in June, 2016. There were 3 subjects were excluded because of left-handed, 1 subject was excluded for big calcification in occipital lobe, 8 subjects were excluded due to losing parts of data during 2 years follow-up. After carefully screening, 135 right-handed cognitively intact elderly (20 APOE ε2 carriers, 41 APOE ε4 carriers and 74 controls) from ADNI cohort whose data met all quality control criteria were included.

According to ADNI manual, the classification of cognitive normal was: the subject had an MMSE between 24-30, and a clinical dementia rating score (CDR) of 0. Additionally, they were non-depressed (score of geriatric depression scale <5). All subjects were screened and excluded for history of obvious head trauma that could impair cognitive function, history of addictions, neurologic or psychiatric disease, or treatments that would damage cognitive function. Notably, subjects in present study were not preselected for presence or absence of WMH. Besides, according to the CDR score and the doctor notes, there was no subjects were converted to dementia during 2 years follow-up. For up-to-date information, see www.adni-info.org.

### Neuropsychological assessment and APOE genotyping

MMSE was performed as a global measure for cognitive performance. Vascular disease was ascertained by self-report, which incorporated current or past diagnosis, treatment of diabetes or hypertension, and whether smoke. The risk of cerebrovascular component was assessed by modified Hachinski Ischemia Score (HIS) [12].

Genotyping of all subjects for APOE allele status was performed using DNA extracted from peripheral blood cells. The cells were collected in 1 EDTA plastic tubes (10 ml) and sent by express mail to the University of Pennsylvania AD Biofluid Bank Laboratory by overnight delivery at room temperature.

### CSF samples and quantification of β-amyloid and tau

CSF was downloaded from the LONI site. Levels of Aβ_1-42_, total tau (t-tau) and phosphorylated tau (p-tau_181_) were measured from CSF samples, which were obtained using the standardized ADNI protocol as previously described [13]. Meanwhile, participants with CSF levels outside 3 SDs above or below the mean were excluded. It should be noted that not all subjects in present study have CSF sample because lumbar puncture is an invasive procedure and not obligatory for healthy subjects. Thus, the final samples for CSF analyses included 16 out of 20 APOE ε2 carriers, 61 out of 74 APOE ε3 homozygotes and 32 out of 41 APOE ε4 carriers.

### Data acquisition

All participants were scanned using a 3.0-Tesla MRI scanner. The 3D MPRAGE T1-weighted sequence were acquired using the following parameters: repetition time (TR) =2300 ms; echo time (TE) =2.98 ms; inversion time (TI) =900 ms; 170 sagittal slices; within plane FOV=256 × 240 mm2; voxel size=1.1×1.1×1.2 mm^3^; flip angle=9°; bandwidth=240 Hz/pix. The T2 FLAIR scans were obtained using an echo-planar imaging sequence with the following parameters: TR= 9000 ms, TE= 90 ms, and TI= 2500 ms. Axial T2*-weighted MRI scans were obtained using gradient echo sequence with the following parameters: TR= 650 ms, TE= 20 ms, Flip angle= 20° number of slices =50, slice thickness =4.0 mm. For PiB-PET, the following imaging parameters were used on a Siemens scanner. Image matrix= 128*128, number of slices=63, pixel resolution=2×2×2 mm^3^; slice thickness =2.4mm; radiopharmaceutical= 11C-PiB, reconstruction method= iterative. For up-to-date information, see www.adni-info.org.

### WMH segmentation and quantification

Our previous WMH segmentation methods were used [14], and we briefly described the method here. For each subject, WMH lesion map was automatically created based on 3D MPRAGE T1-weighted and T2 FLAIR image using Lesion Segmentation Toolbox [15]. The masks were then manually corrected by two experienced neuroradiologist (MMZ, HPY). Then we coregistered the 3D MPRAGE T1, T2 FLARI and corrected masks to the standard atlas (UNC adult brain atlas template, www.nitrc.org). After combining our corrected masks with the UNC lobar parcellation mask, we separated WMH ROI into the 4 brain lobes (frontal, occipital, temporal and parietal lobe). Finally, WMH volume in each brain lobe was calculated by multiplying voxel numbers in each lobar ROI by voxel size.

### Rating of dPVS, lacune and MBs

**Figure 3:**
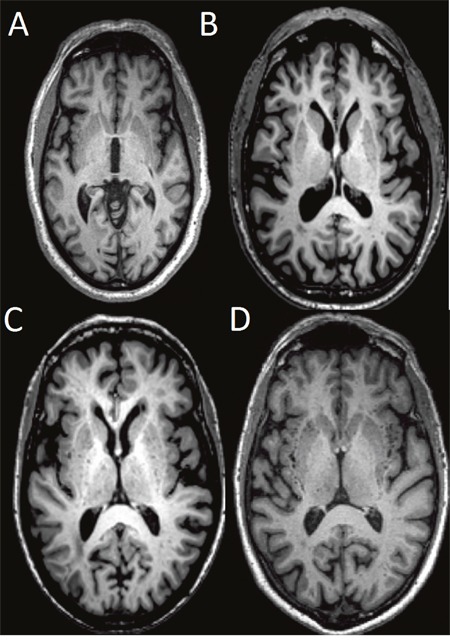
Severity scores of dPVS in the BG Specifically, dPVS severity is rated 1 when there were <5 dPVS **(A)**, rated 2 when there were 5~10 dPVS **(B)**, rated 3 when there were >10 dPVS but the number is still countable **(C)**, and rated 4 when the number is uncountable **(D)**.

**Figure 4:**
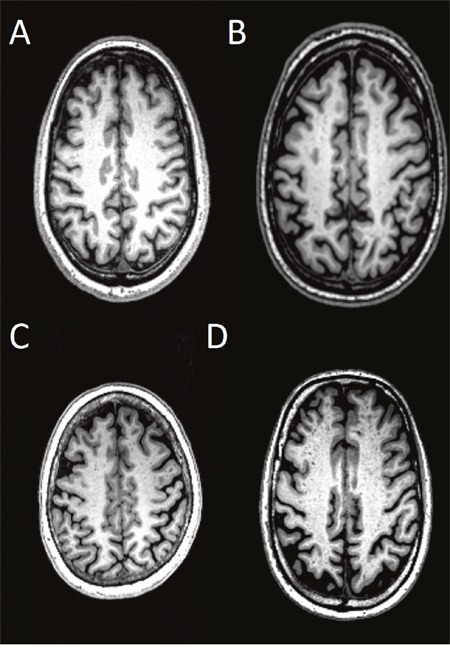
Severity scores of dPVS in the WM Specifically, dPVS severity is rated 1 when there were <10 dPVS in total **(A)**, rated 2 when there were >10 PVS in total but no more than 10 dPVS in a single section **(B)**, rated 3 when there were 10~20 dPVS in the section containing the greatest number of dPVS **(C)**, and rated 4 when there were >20 dPVS in any single section **(D)**.

High-resolution 3D MPRAGE T1-weighted structural data was used for the assessment of dPVS [16, 17]. In general, dPVS was defined as CSF-like signal lesions (hypointense on T1 and hyperintense on T2) of round, ovoid, or linear shape with a maximum diameter <3 mm, having smooth delineated contours [1]. The severity is rated based on the number of dPVS, and dPVS in basal ganglia (BG) and cerebral WM are rated separately. The detailed rating are: in basal ganglia, dPVS severity is rated 1 when there were <5 dPVS, rated 2 when there were 5~10 dPVS, rated 3 when there were >10 dPVS but the number is still countable, and rated 4 when the number is uncountable (Figure 3); in cerebral white matter (WM), dPVS severity is rated 1 when there were <10 dPVS in total, rated 2 when there were >10 PVS in total but no more than 10 dPVS in a single section, rated 3 when there were 10~20 dPVS in the section containing the greatest number of dPVS, and rated 4 when there were >20 dPVS in any single section [17] (Figure 4).

Lacune was defined as CSF-like hypointensity with surrounding rim of hyperintensities in T2-FLAIR data, of between 3 mm and 15 mm in diameter. Lacune was recorded as present if there was at least 1 Lacune lesion visible, or absent if there was no Lacune lesion visible. MBs were defined as areas of signal void on T2*-weighted MRI. The number of MBs in whole cerebral was counted and recorded [1, 7].

Two experienced neuroradiologist (MM.Z and XJ.X) who were blind to the patients’ clinical data and follow-up status rated the subjects. In case of dispute a decision was made on the basis of the third experienced neuroradiologist (PY. H).

### Regional assessment of PiB-PET data

Detailed methods have been previously described for acquisition and processing of PiB-PET scans for the ADNI sample, and were briefly reported below [18]. Regional normalized PiB standardized uptake values (SUV) used in this analysis were obtained from a dataset generated by the University of Pittsburgh as described briefly below. First, each subject's PET image is coregistered using SPM 8 to that subject's MRI TIWI data. Second, the FreeSurfer processing was performed in skull-strip, segment and delineate cortical and subcortical regions in all MRI scans. Then, each subject's PIB image was sampled with an automated 14 ROIs template including 9 cortical and 3 subcortical ROIs as well as the cerebellum. Finally, according to research purpose, we merely extracted the frontal, parietal, occipital and temporal PIB SUV measures as continuous variables. The SUV of PET is normalized by the mean SUV of the cerebellum reference region. Further information on the post processing steps of the PiB-PET scans by the University of Pittsburgh can be found at http://adni.loni.ucla.edu/research/pet-post-processing/.

### Statistical analyses

The group differences between age, education, Aβ_1-42_ concentration, t-tau, p-tau_181_, WMH volume, dPVS, MB and lacune at baseline were performed by one-way analysis of variance (ANOVA). The post-hoc pair wise T-tests were performed if the differences were significant (p<0.05, corrected by Bonferroni). In case of the group differences of dPVS, MB, and lacune, non-parametric K-W test was used. The progression of WMH volume in 3 time points were tested with ANOVA of repeated measures data, APOE genotype were entered to the model as between-subjects factor.

The correlation analyses between Aβ_1-42_ concentration and CSVD, as well as between cognition and CSVD were performed by Pearson or Spearman correlation test as appropriate. A first General lineal model (GLM) was used to determine the effect of APOE genotype and Aβ_1-42_ concentration on WMH volume. In this model, WMH volume was the dependent variable and APOE genotype is the fix factor, and Aβ_1-42_ concentration were added as the co-variants. A second GLM was used to study predictive variables for progression of MMSE during follow-up. APOE ε4 and ε2 allele carrying status were added as fixed factor. Variables added to the model included: the markers of CSVD (WMH volume, dPVS ranking, MB, lacune) that showed significant group differences, age, sex and high blood pressure score. All 2-way interaction between variables was tested in the model as well.

## Abbreviations

APOE: apolipoprotein E
CSVD: cerebral small-vascular disease
WMH: white matter hyperintensities
dPVS: dilated perivascular space
MBs: microbleeds

## References

[1] Wardlaw JM, Smith EE, Biessels GJ, Cordonnier C, Fazekas F, Frayne R, Lindley RI, O'Brien JT, Barkhof F, Benavente OR (2013). Neuroimaging standards for research into small vessel disease and its contribution to ageing and neurodegeneration. Lancet Neurol.

[2] Pantoni L (2010). Cerebral small vessel disease: from pathogenesis and clinical characteristics to therapeutic challenges. Lancet Neurol.

[3] Kivipelto M, Helkala EL, Laakso MP, Hänninen T, Hallikainen M, Alhainen K, Soininen H, Tuomilehto J, Nissinen A (2001). Midlife vascular risk factors and Alzheimer's disease in later life: longitudinal, population based study. BMJ.

[4] Dichgans M, Zietemann V (2012). Prevention of vascular cognitive impairment. Stroke.

[5] Brickman AM, Siedlecki KL, Muraskin J, Manly JJ, Luchsinger JA, Yeung LK, Brown TR, Decarli C, Stern Y (2011). White matter hyperintensities and cognition: Testing the reserve hypothesis. Neurobiology of Aging.

[6] Stampfer MJ (2006). Cardiovascular disease and Alzheimer's disease: common links. J Intern Med.

[7] Lyall DM, Muñoz Maniega S, Harris SE, Bastin ME, Murray C, Lutz MW, Saunders AM, Roses AD (2015). Valdés Hernández MDC, Royle NA. APOE/TOMM40 genetic loci, white matter hyperintensities, and cerebral microbleeds. Int J Stroke.

[8] Kim J, Basak JM, Holtzman DM (2009). The role of apolipoprotein E in Alzheimer's disease. Neuron.

[9] Suri S, Heise V, Trachtenberg AJ, Mackay CE (2013). The forgotten APOE allele: a review of the evidence and suggested mechanisms for the protective effect of APOE ɛ2. Neurosci Biobehav Rev.

[10] Paternoster L, Chen W, Sudlow CL (2009). Genetic determinants of white matter hyperintensities on brain scans. Stroke.

[11] Schilling S, Destefano AL, Sachdev PS, Choi SH, Mather KA, Decarli CD, Wen W, Høgh P, Raz N, Au R (2013). APOE genotype and MRI markers of cerebrovascular disease: systematic review and meta-analysis. Neurology.

[12] Hachinski VC, Iliff LD, Zilhka E, Boulay GH, Mcallister VL, Marshall J, Russell RW, Symon L (1975). Cerebral blood flow in dementia. Neurology.

[13] Shaw LM, Vanderstichele H, Knapik-Czajka M, Figurski M, Coart E, Blennow K, Soares H, Simon AJ, Lewczuk P, Dean RA (2011). Qualification of the analytical and clinical performance of CSF biomarker analyses in ADNI. Acta Neuropathol.

[14] Luo X, Jiaerken Y, Yu X, Huang P, Qiu T, Jia Y, Sun J, Zhou J, Zhang M (2016). Alzheimer's Disease Neuroimaging Initiative (ADNI). Affect of APOE on information processing speed in non-demented elderly population: a preliminary structural MRI study. Brain Imaging Behav.

[15] Schmidt P, Gaser C, Arsic M, Buck D, Förschler A, Berthele A, Hoshi M, Ilg R, Schmid VJ, Zimmer C (2011). An automated tool for detection of FLAIR-hyperintense white-matter lesions in multiple sclerosis. Neuroimage.

[16] Chen W, Song X, Zhang Y (2011). Alzheimer's Disease Neuroimaging Initiative. Assessment of the Virchow-Robin Spaces in Alzheimer disease, mild cognitive impairment, and normal aging, using high-field MR imaging. AJNR Am J Neuroradiol.

[17] Zhu YC, Dufouil C, Mazoyer B, Soumaré A, Ricolfi F, Tzourio C, Chabriat H (2011). Frequency and location of dilated Virchow-Robin spaces in elderly people: a population-based 3D MR imaging study. AJNR Am J Neuroradiol.

[18] Jagust WJ, Bandy D, Chen K, Foster NL, Landau SM, Mathis CA, Price JC, Reiman EM, Skovronsky D, Koeppe RA (2010). The Alzheimer's Disease Neuroimaging Initiative positron emission tomography core. Alzheimers Dement.

[19] Myers R, Schaefer E, Wilson P, d'Agostino R, Ordovas J, Espino A, Au R, White R, Knoefel J, Cobb J (1996). Apolipoprotein E element 4 association with dementia in a population-based study The Framingham Study. Neurology.

[20] Payabvash S, Souza LC, Wang Y, Schaefer PW, Furie KL, Halpern EF, Gonzalez RG, Lev MH (2011). Regional ischemic vulnerability of the brain to hypoperfusion: the need for location specific computed tomography perfusion thresholds in acute stroke patients. Stroke.

[21] Raz N, Rodrigue KM, Acker JD (2003). Hypertension and the brain: vulnerability of the prefrontal regions and executive functions. Behav Neurosci.

[22] Wen W, Sachdev P (2004). The topography of white matter hyperintensities on brain MRI in healthy 60-to 64-year-old individuals. Neuroimage.

[23] Jiang Q, Lee CD, Mandrekar S, Wilkinson B, Cramer P, Zelcer N, Mann K, Lamb B, Willson TM, Collins JL (2008). ApoE promotes the proteolytic degradation of Aβ. Neuron.

[24] Reinvang I, Espeseth T, Westlye LT (2013). APOE-related biomarker profiles in non-pathological aging and early phases of Alzheimer's disease. Neurosci Biobehav Rev.

[25] Gottlieb DJ, DeStefano A, Foley D, Mignot E, Redline S, Givelber R, Young T (2004). APOE epsilon4 is associated with obstructive sleep apnea/hypopnea: the Sleep Heart Health Study. Neurology.

[26] Xie L, Kang H, Xu Q, Chen MJ, Liao Y, Thiyagarajan M, O’Donnell J, Christensen DJ, Nicholson C, Iliff JJ (2013). Sleep drives metabolite clearance from the adult brain. Science.

[27] Iliff JJ, Wang M, Liao Y, Plogg BA, Peng W, Gundersen GA, Benveniste H, Vates GE, Deane R, Goldman SA (2012). A paravascular pathway facilitates CSF flow through the brain parenchyma and the clearance of interstitial solutes, including amyloid β. Sci Transl Med.

[28] Plog BA, Dashnaw ML, Hitomi E, Peng W, Liao Y, Lou N, Deane R, Nedergaard M (2015). Biomarkers of traumatic injury are transported from brain to blood via the glymphatic system. J Neurosci.

[29] Peng W, Achariyar TM, Li B, Liao Y, Mestre H, Hitomi E, Regan S, Kasper T, Peng S, Ding F (2016). Suppression of glymphatic fluid transport in a mouse model of Alzheimer's disease. Neurobiology of disease.

[30] Kalaria RN (1997). Cerebrovascular degeneration is related to amyloid-β protein deposition in alzheimer's disease. Ann N Y Acad Sci.

[31] Promjunyakul N, Lahna D, Kaye J, Dodge H, Erten-Lyons D, Rooney W, Silbert L (2015). Characterizing the white matter hyperintensity penumbra with cerebral blood flow measures. Neuroimage Clin.

[32] de Groot M, Verhaaren BF, de Boer R, Klein S, Hofman A, van der Lugt A, Ikram MA, Niessen WJ, Vernooij MW (2013). Changes in normal-appearing white matter precede development of white matter lesions. Stroke.

[33] Maillard P, Fletcher E, Harvey D, Carmichael O, Reed B, Mungas D, DeCarli C (2011). White matter hyperintensity penumbra. Stroke.

[34] PPromjunyakul NO, Lahna DL, Kaye JA, Dodge HH, Erten-Lyons D, Rooney WD, Silbert LC (2016). Comparison of cerebral blood flow and structural penumbras in relation to white matter hyperintensities: a multi-modal magnetic resonance imaging study. J Cereb Blood Flow Metab.

[35] Burton EJ, Kenny RA, O’Brien J, Stephens S, Bradbury M, Rowan E, Kalaria R, Firbank M, Wesnes K, Ballard C (2004). White matter hyperintensities are associated with impairment of memory, attention, and global cognitive performance in older stroke patients. Stroke.

[36] Chen YF, Wang H, Chu Y, Huang YC, Su MY (2006). Regional quantification of white matter hyperintensity in normal aging, mild cognitive impairment, and Alzheimer's disease. Dement Geriatr Cogn Disord.

[37] Bonelli RM, Cummings JL (2007). Frontal-subcortical circuitry and behavior. Dialogues Clin Neurosci.

[38] Heyder K, Suchan B, Daum I (2004). Cortico-subcortical contributions to executive control. Acta Psychol (Amst).

[39] Charidimou A, Meegahage R, Fox Z, Peeters A, Vandermeeren Y, Laloux P, Baron J-C, Jäger HR, Werring DJ (2013). Enlarged perivascular spaces as a marker of underlying arteriopathy in intracerebral haemorrhage: a multicentre MRI cohort study. J Neurology Neurosurg Psychiatry.

[40] Hurford R, Charidimou A, Fox Z, Cipolotti L, Jager R, Werring DJ (2014). MRI-visible perivascular spaces: relationship to cognition and small vessel disease MRI markers in ischaemic stroke and TIA. J Neurology Neurosurg Psychiatry.

[41] Potter GM, Doubal FN, Jackson CA, Chappell FM, Sudlow CL, Dennis MS, Wardlaw JM (2015). Enlarged perivascular spaces and cerebral small vessel disease. Int J Stroke.

[42] Rouhl R, Van Oostenbrugge R, Knottnerus I, Staals J, Lodder J (2008). Virchow-Robin spaces relate to cerebral small vessel disease severity. J Neurol.

[43] Doubal FN, MacLullich AM, Ferguson KJ, Dennis MS, Wardlaw JM (2010). Enlarged perivascular spaces on MRI are a feature of cerebral small vessel disease. Stroke.

[44] Zhu YC, Dufouil C, Soumaré A, Mazoyer B, Chabriat H, Tzourio C (2010). High degree of dilated Virchow-Robin spaces on MRI is associated with increased risk of dementia. J Alzheimers Dis.

[45] Viswanathan M, Salman RA, Steven Warach M, Lenore J (2009). Cerebral microbleeds: a field guide to their detection and interpretation. Lancet Neurol.

